# A retrospective, cross-sectional study of real-world values of cardiovascular risk factors using a healthcare database in Japan

**DOI:** 10.1186/1471-2261-14-120

**Published:** 2014-09-17

**Authors:** Daisuke Shima, Yoichi Ii, Yuji Yamamoto, Satoshi Nagayasu, Yumiko Ikeda, Yoko Fujimoto

**Affiliations:** Medical Affairs, Pfizer Japan Inc, 3-22-7 Yoyogi, Shibuya-ku, Tokyo, 151-8589 Japan; Clinical Statistics, Pfizer Japan Inc, 3-22-7 Yoyogi, Shibuya-ku, Tokyo, 151-8589 Japan; MinaCare Co. Ltd., Japan, 1-23 Kandanishiki-cho, Chiyoda-ku, Tokyo, 101-0054 Japan; Medical Writing, Pfizer Japan Inc, 3-22-7 Yoyogi, Shibuya-ku, Tokyo, 151-8589 Japan

**Keywords:** National Health and Nutrition Survey, Specific health checkups and specific health guidance, MinaCare database, Noncommunicable disease, Japanese epidemiology, Blood pressure, Low-density lipoprotein cholesterol, Hemoglobin A1c

## Abstract

**Background:**

Data collected by the Japanese Ministry of Health, Labour and Welfare (MHLW), namely data from the Specific Health Checkups and Specific Health Guidance (MHLW-SH) and the National Health and Nutrition Survey (MHLW-H&N) allow assessment of blood pressure (BP), low-density lipoprotein cholesterol (LDL-C), and hemoglobin A1c (HbA1c) in Japan. Recently, a large database of employment-based health insurance has been developed by MinaCare Co. Ltd.

**Methods:**

A retrospective, cross-sectional study using the Japanese healthcare checkup database developed by MinaCare Co. Ltd. was designed to investigate the distribution of real-world values of BP, LDL-C, and HbA1c in Japan. Data in the MinaCare database were also compared with those in the two national data sources to assess the extent to which the health status in Japan is reflected in each data source.

**Results:**

Of the healthcare checkup results of 232515 subjects in the 2011 MinaCare database, 49.9% were male and 50.1% were female. The age of the subjects ranged from <20 to >70 years. The proportion of subjects with systolic BP (SBP) ≥140 mmHg, LDL-C ≥140 mg/dL, and HbA1c ≥6.1% generally increased with increasing age. If one focused on the upper-end age group representing the majority of the MinaCare study population (i.e. age range, 55–59 years), the proportions of subjects with SBP ≥140 mmHg, LDL–C ≥140 mg/dL, and HbA1c ≥6.1% were 19.0%/12.2% (males/females), 27.2%/42.7%, and 13.5%/5.4%, respectively. The MinaCare database was mostly comparable with the two national data sources; however, some notable differences in BP and lipid parameters were found between MHLW-H&N and the other two data sources.

**Conclusions:**

Analysis of the MinaCare database indicated that a substantial proportion of subjects did not achieve the target BP, LDL-C, and HbA1c levels to reduce the risk of future cardiovascular and cerebrovascular disease events. The results were generally consistent with those of the national data sources. Considering its characteristics of low selection bias, large sample size, wide age distribution, and high flexibility in analysis of subject-level data, the MinaCare database is highly valuable for studying the health status of the population covered by employment-based health insurance.

## Background

Cardiovascular and cerebrovascular diseases (CVDs) are the second and third leading causes of death in Japan, and it is well recognized that hypertension (HT), hyperlipidemia, and diabetes mellitus are the major risk factors for CVDs on a global basis [1, 2]. The status of HT, hyperlipidemia, and diabetes mellitus is monitored by measuring blood pressure (BP), low-density lipoprotein cholesterol (LDL-C), and hemoglobin A1c (HbA1c) levels, respectively, which also indicate the future probability of CVDs [1–3]. Therefore, academic societies provide target values for these parameters to achieve better management and prevention of these diseases worldwide. Such target values are also provided by Japanese Society of Hypertension (JSH) [4], the Japan Atherosclerosis Society (JAS) [5], and the Japan Diabetes Society (JDS) [6] for Japanese populations.

To monitor the actual health status of Japanese residents and to promote their health, the Japanese Ministry of Health, Labour and Welfare (MHLW) regularly conducts the National Health and Nutrition Survey (MHLW-H&N) [7] and Specific Health Checkups and Specific Health Guidance (MHLW-SH) [8, 9].

MHLW-H&N has been conducted annually since 1947 to monitor and obtain basic information on health, nutrition, and life style to further enhance measures to improve the overall health of Japanese residents [7]. Data is collected from Japanese residents of 1 year and older who are randomly selected from each of approximately 2000 geographical sampling units and agree to participate in the survey. This data source has a relatively small sample size (6914 subjects in 2011).

MHLW-SH was initiated in 2008 under the Japanese health insurance system [8, 9]. The health insurance system has provided universal coverage and nation-wide cost sharing since 1961. By law, insurers must offer their subscribers aged 40–74 years an annual health checkup that includes blood chemistry, urinalysis, and BP measurement. A total of 22232094 subjects underwent the annual checkup in 2010. The sample size of MHLW-SH data is thus much larger than that of MHLW-H&N data, but it does not contain data of individuals aged <40 years. The individuals included in the MHLW-SH data source also represent less than half of the total targeted population (42.6% in 2010) because annual checkups are voluntary and rely on individuals’ voluntary participation. This could cause significant selection bias.

These survey results are open to the public, and the health status of the Japanese population is often estimated based on these results. However, because access to the subject-level data from these data sources is limited, researchers often cannot conduct analyses that would answer original research questions. Taking into consideration the limitations of these two data sources, such as the selection bias of patients, limited number of patients, limited age distribution, and limited access to subject-level data [10], we believe that an alternative and/or supplemental database is necessary to investigate and understand the actual health status of this population.

Since 2011, a large database containing the results of employment-based health insurance healthcare checkups has been developed by MinaCare Co. Ltd (Tokyo, Japan).

The present study was conducted to better understand the health status of working individuals and their codependents in Japan by analyzing BP, LDL-c, and HbA1c data included in the MinaCare database. To investigate real-world values from various perspectives and to assess the extent to which the actual health status in Japan is reflected in each data source, the analysis results of data from the MinaCare database were compared with those from the publicly available summaries of MHLW-SH and MHLW-H&N data. The usefulness of the MinaCare database was also discussed.

## Methods

### Study design

This was a retrospective, cross-sectional study using a commercially available Japanese healthcare database (MinaCare Co. Ltd., Tokyo, Japan). The main aim of the study was to examine BP, LDL-c and HbA1c values from health checkup data from a cross-sectional viewpoint, where a single time point for each individual was selected for analysis. Smoking status, body mass index (BMI), and waist circumference were also evaluated, because these three measures are also known risk factors for CVDs. MinaCare data were then compared with the latest (at the time of analysis) publicly available summary results from MHLW-SH (2010) [8] and MHLW-H&N (2011) [7].

### MinaCare database

**Figure 1:**
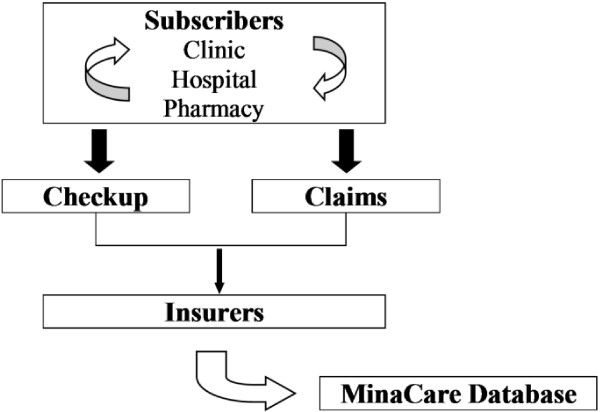
**Schematic description of the MinaCare database.** The MinaCare database consists of checkup and claim data provided by employment-based health insurance in Japan since 2010.

MinaCare Co. Ltd. manages healthcare data provided by employment-based health insurance and offers health risk reduction plans to corporate employees diagnosed with obesity or those who have a high risk for any lifestyle-related diseases. The MinaCare database includes regularly updated data of checkups and medical and pharmaceutical claims since 2010 (Figure 1). The population covered by the database includes working individuals and their dependent family members, and the database covers a wide range of age groups. Employment-based insurance covers large-scale, nation-wide retailers, manufacturers, and those in food, information, transportation and energy industries. Therefore, the MinaCare database includes health information of individuals with minimal geographic or occupational bias, but it has the limitation that individuals in the primary industries such as agriculture, fishery and forestry, and those who are self-employed are not included. Health checkup data include information on subjects’ demographics, smoking status, vital signs, clinical laboratory test data, and administrative information. Data of medical and pharmaceutical claims from approximately 1.1 million subjects that are 3%-4% of the total insured individuals in Japan, and health checkup data from approximately 338000 subjects were collected into the MinaCare database as of November 2013. Considering the nature of MinaCare Co. Ltd, which manages healthcare data provided by employment-based health insurance, and the fact that insurers must offer subscribers an annual health checkup, it seems obvious that a high percentage of the target population undergoes annual checkups.

### Anonymization of subjects

This study used subject-level electronic health related databases that protected the identity of individuals. MinaCare is allowed to use such anonymized data under the data transfer contract with its client health insurers. We referred the “Ethical Guidelines for Epidemiological Research” laid down by the Japanese Government [11], although this study is outside the scope because the data is protected by anonymization.

### Data extraction and management of irregular data

All health checkup data included in the MinaCare database for the fiscal years from 2010 to 2012 were extracted. Health checkup examinations are normally conducted annually, thus this dataset included subjects with examination years that ranged from 1 up to a maximum of 3 years. In this study, all subjects who had at least one set of health checkup data for the fiscal year 2011 were selected (a total of 232515 subjects). For a few subjects who underwent multiple examinations in the same year, earliest examination data were used. It was noted that 2011 was a representative cross-sectional year for the range of fiscal years from 2010 to 2012 and was also a single year that was most comparable with the timing of the two national surveys.

Missing values were not imputed and were excluded from analysis, unless otherwise noted. Non-numeric or impossible values for numeric variables were handled as missing values and were also excluded from the summary. The proportion and pattern of missing values were monitored to consider any implications in the interpretation of the results.

### Study variables

General information (subject ID, year of examination, birth year/month, and date of examination), sex, body size measurements (height, weight, waist circumference, BMI, obesity score), smoking status, vital signs (average BP), and clinical laboratory tests [fasting blood glucose (FBG), HbA1c, urine sugar, uric protein, total cholesterol, triglyceride (TG), high density lipoprotein cholesterol (HDL-c), and LDL-c] data were extracted from the database. In addition, the age at the time of examination was computed from the birth year/month by imputing the first day of the month for the birth date. Smoking status was based on a subject questionnaire regarding the subject’s current smoking status.

In terms of HbA1c values, the values of the Japan Diabetes Society (JDS) were used in Japan in 2011 for health checkups. HbA1c (JDS) ≥6.1%, comparable with HbA1c [National glycohemoglobin standardization program (NGSP)] ≥6.5%, was the diagnostic criterion for diabetes mellitus based on the JDS guideline [6, 12].

### Study endpoints

The primary endpoints of this study were the distribution of demographic variables, the distribution of BP [systolic BP (SBP) and diastolic BP (DBP)], LDL-c, and HbA1c values, and the proportion of subjects who achieved the predefined BP, LDL-c, and HbA1c cutoff levels. These endpoints were examined in various subgroups of interest.

The secondary endpoints of this study included the distributions of HDL-c, TG and FBG values and the proportion of subjects who achieved the respective predefined cutoff levels and the proportion of subjects within each cardiovascular and cerebrovascular risk level. These endpoints were examined in various subgroups of interest.

Cutoff levels for BP (SBP and DBP), lipid parameters (LDL-c, HDL-c, and TG), HbA1c and FBG were based on the JSH 2009 guideline [4], JAS 2012 guideline [5] and JDS 2013 guideline [6], respectively.

Age (at examination), sex, height, weight, BMI, waist circumference, and smoking status were considered as covariates. Subgroups defined by the levels of the covariates were examined.

### Statistical method

**Figure 2:**
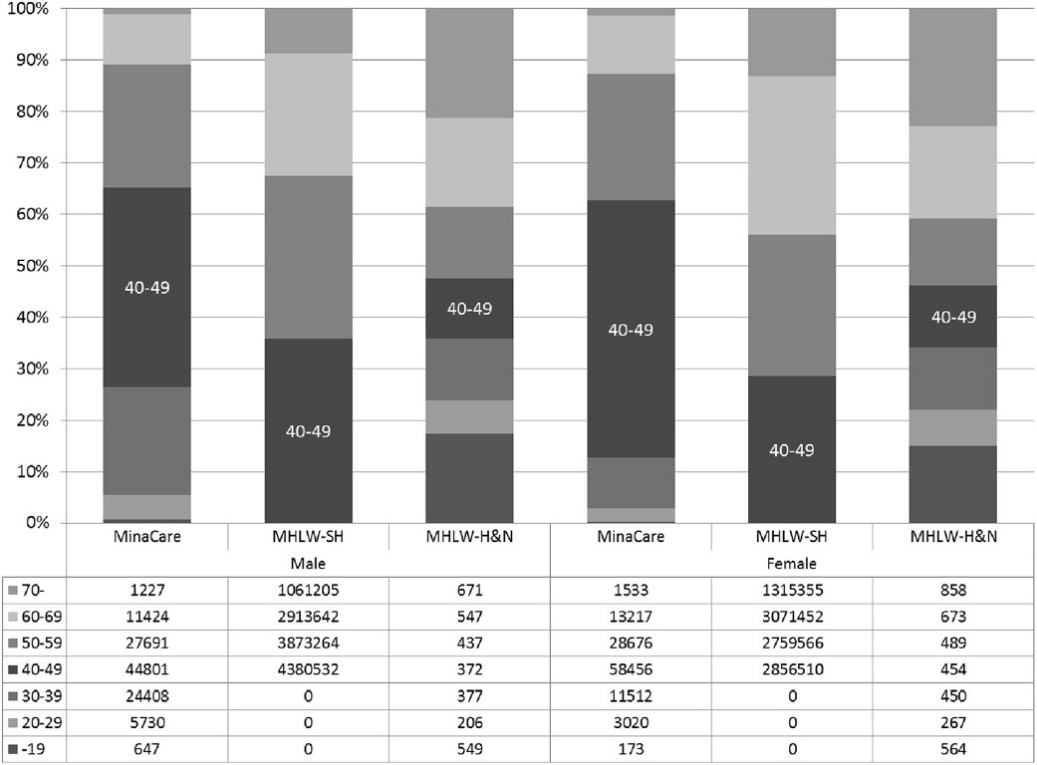
**Age distributions in the MinaCare, MHLW-SH, and MHLW-H&N data.**

**Figure 3:**
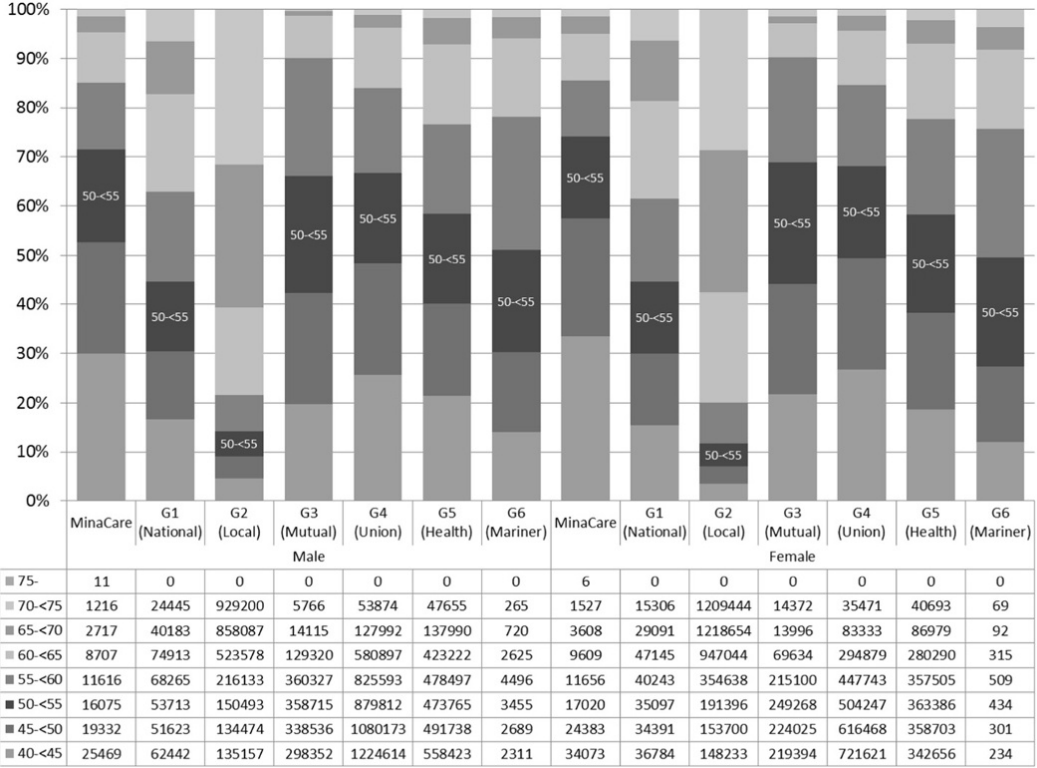
**Age distributions in the MinaCare and MHLW-SH data.** The age distributions of subjects ≥40 years are shown and the MHLW-SH data are classified by employment-based health insurance.

This analysis was primarily based on descriptive statistical methods, and formal statistical inference was not used. For continuous data, summary statistics such as mean, median, minimum, maximum, and standard deviation (SD) were used. For binary and categorical data, summary statistics such as numbers and proportions (percentages) were used. Graphical presentations were used to describe data. To enhance visual comparisons, graphical presentations of means and proportions of various endpoints were made without standard errors or confidence intervals. Because the number of subjects (n) in each sex and age category was generally very large (as seen in Figures 2 and 3), this simplification did not affect the appropriate interpretation of data. However, special notes on variability were made when indicated.

The Structured Query Language was used to extract data from the MinaCare database. SAS version 9.2 was used to derive datasets for statistical analyses. R (version 2.15.2) and MS EXCEL 2007 were used to create graphs.

### Existing data sources used for the comparison with MinaCare database

The summary level results of MHLW-SH data (2010) and MHLW-H&N data (2011) were obtained from existing reports open to the public on the MHLW homepage [7, 8]. The number of reported subjects (sex tabulation) was 22232094 (12228976 males and 10003118 females) in MHLW-SH data (2010) and 6914 (3159 males and 3755 females) in MHLW-H&N data (2011).

### Classification of adult BP values by the JSH 2009 guideline

Subjects were classified according to their BP levels based on the JSH 2009 guideline [4]. In this guideline, the following six categories were defined based on combination of SBP and DBP values: ideal, normal, normal (high), HT class I, HT class II, and HT class III.

## Results

### MinaCare database

#### Overall characteristics of the study population

The characteristics of the study population by sex are shown in Table 1.

**Table 1:** **Characteristics of the study population in the MinaCare database**

	Male	Female
	N = 115928	N = 116587
**Sample size (Total: 232515)**	**115928**	**116587**
**Age (years)**		
n (%) [n missing]	115928 (100) [0]	116587 (100) [0]
−19	647 (0.6)	173 (0.1)
20-29	5730 (4.9)	3020 (2.6)
30-39	24408 (21.1)	11512 (9.9)
40-49	44801 (38.6)	58456 (50.1)
50-59	27691 (23.9)	28676 (24.6)
60-69	11424 (9.9)	13217 (11.3)
70-	1227 (1.1)	1533 (1.3)
Height (cm)		
n [n missing]	115864 [64]	116490 [97]
mean (SD)	170.9 (5.89)	157.8 (5.51)
Weight (kg)		
n [n missing]	115855 [73]	116480 [107]
mean (SD)	69.6 (10.88)	54.4 (9.14)
BMI (kg/m^2^)		
n [n missing]	115851 [77]	116475 [112]
mean (SD)	23.8 (3.37)	21.8 (3.54)
Waist Circumference (cm)		
n [n missing]	114777 [1151]	114486 [2101]
mean (SD)	83.9 (9.02)	77.9 (9.51)
Smoking Status n (%)		
n [n missing]	115105 (100) [823]	115661 (100) [926]
Yes	38425 (33.4)	17616 (15.2)
No	76680 (66.6)	98045 (84.8)

n: number of subjects with available data; n missing: number of subjects with missing data; SD: standard deviation: BMI: body mass index.

**Figure 4:**
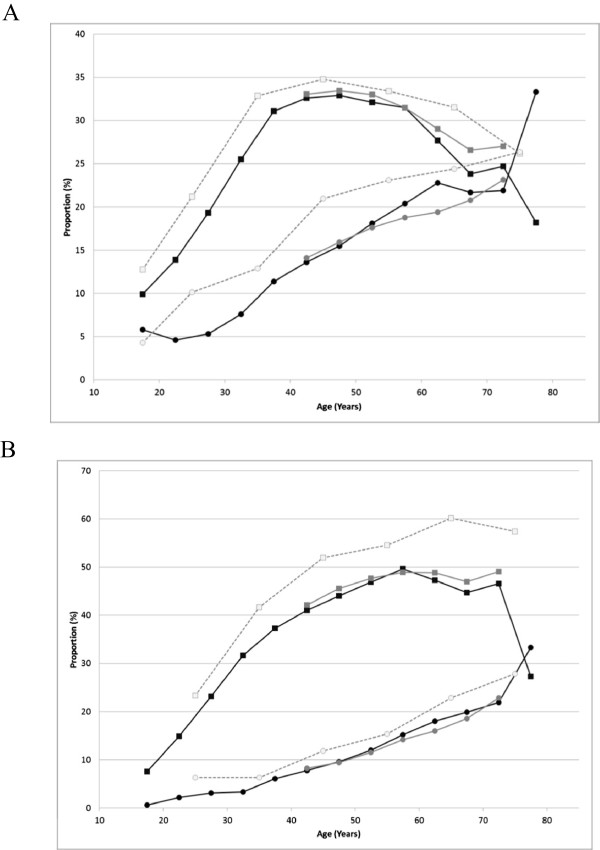
**Distributions of BMI and waist circumference in the MinaCare, MHLW-SH, and MHLW-H&N data. A**: Proportions of subjects with BMI ≥25 (kg/m^2^). **B**: Proportions of subjects with waist circumference >85 cm (male) / >90 cm (female). Squares represent males and circles represent females. Black, grey, and open symbols represent the MinaCare, MHLW-SH, and MHLW-H&N data, respectively. Interpretation of the results for subjects aged ≥75 years requires care due to the small sample size (11 males and 6 females) in the MinaCare data.

**Figure 5:**
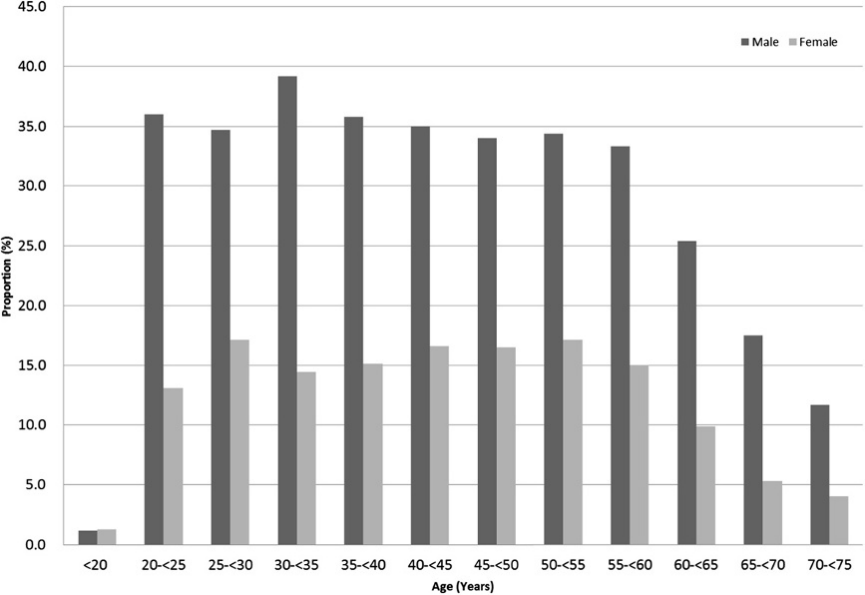
**Proportion of current smokers among males and females by age in the MinaCare data.**

Of 232515 subjects, 49.9% were male and 50.1% were female. The age of subjects ranged from <20 to >70 years (Table 1 and Figure 2). The greatest proportion of the population was in their 40’s in both males (38.6%) and females (50.1%) (Table 1); the proportion in their 40s was nearly 10% higher in female subjects, as shown in Figure 2. Nearly 90% were <60 years in both males and females. When the proportion of subjects with BMI ≥25 kg/m^2^ was plotted against age, the peak age was 40–49 years in males, whereas the proportion gradually increased and plateaued at 60 years or older in females (Figure 4A). Similar trends were seen for the mean BMI values (data not shown). The proportion of subjects with a waist circumference above the cut-off values (85 cm in males and 90 cm in females) gradually increased and plateaued at 60 years or older in males, whereas a steady increase across the entire age range was seen in females (Figure 4B). The proportion of current smokers among males was approximately twice the proportion among females (Figure 5). We noted that age groups were confounded by birth-year cohorts, and any description of “trends with increasing age” in this paper should be interpreted keeping this in mind.

#### BP (SBP and DBP), lipid parameters (LDL-c, HDL-c, and TG), HbA1c and FBG levels

Summary statistics of BP, lipid parameters, HbA1c, and FBG values are presented in Table 2.

**Table 2:** **BP, lipid parameters, HbA1c and FBG levels in the study population in the MinaCare database**

	Male	Female
	N = 115928	N = 116587
SBP (mmHg)		
n [n missing]	115590 [338]	116363 [224]
mean (SD)	121.8 (15.25)	114.4 (16.54)
DBP (mmHg)		
n [n missing]	115586 [342]	116360 [227]
mean (SD)	77.1 (11.29)	70.3 (11.22)
LDL-c (mg/dL)		
n [n missing]	115188 [740]	114378 [2209]
median (1st; 3rd quartiles)	120 (101; 141)	116 (96; 138)
HDL-c (mg/dL)		
n [n missing]	115228 [700]	114460 [2127]
median (1st; 3rd quartiles)	55 (47; 65)	69 (59; 79)
TG (mg/mL)		
n [n missing]	115222 [706]	114454 [2133]
median (1st; 3rd quartiles)	105 (74; 155)	72 (53; 101)
HbA1c (% [JDS])		
n [n missing]	79663 [36265]	98308 [18279]
median (1st; 3rd quartiles)	5.0 (4.8; 5.3)	5.0 (4.8; 5.2)
FBG (mg/dL)		
n [n missing]	112350 [3578]	105129 [11458]
median (1st; 3rd quartiles)	93 (87; 100)	89 (84; 95)

n: number of subjects with available data; n missing: number of subjects with missing data; SD: standard deviation; SBP/DBP: systolic/diastolic blood pressure; LDL-c/HDL-c: low/high density lipoprotein cholesterol; TG: triglyceride; HbA1c: hemoglobin A1c; JDS: Japan Diabetes Society; FBG: free blood glucose.

**Figure 6:**
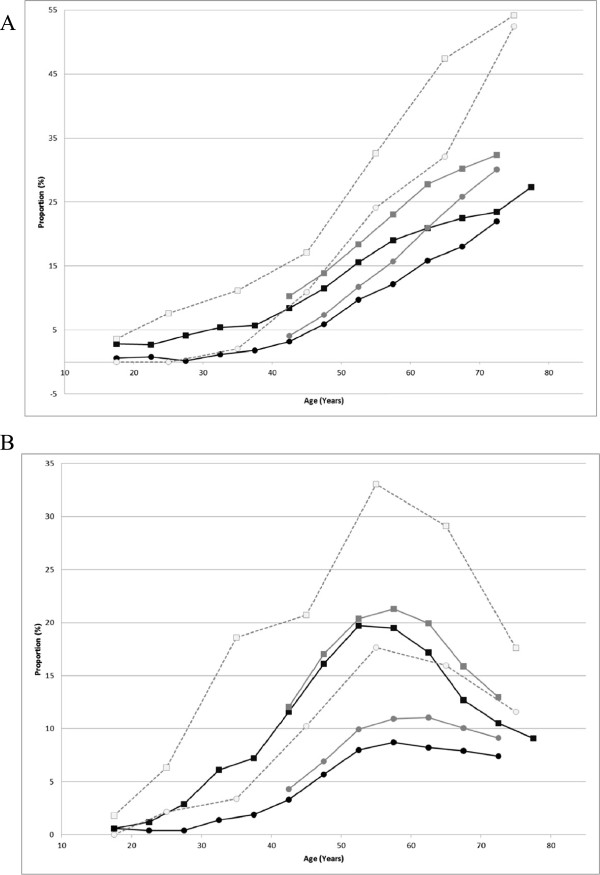
**BP distributions in the MinaCare, MHLW-SH, and MHLW-H&N data. A**: Proportions of subjects with SBP ≥140 mmHg. **B**: Proportions of subjects with DBP ≥90 mmHg. Squares represent males and circles represent females. Black, grey, and open symbols represent the MinaCare, MHLW-SH, and MHLW-H&N data, respectively. Interpretation of the results for subjects aged ≥75 years requires care due to the small sample size (11 males and 6 females) in the MinaCare data.

**Figure 7:**
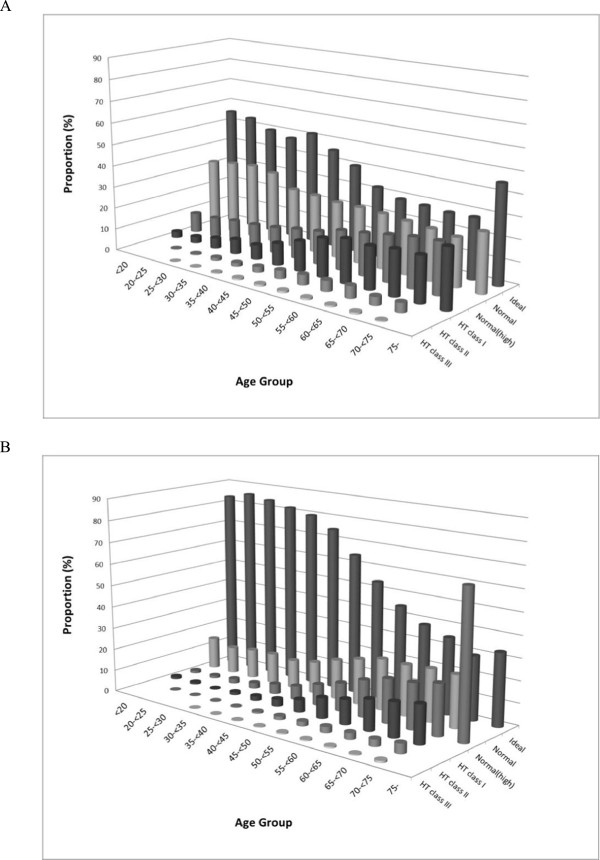
**Proportions of subjects in each BP classification category (JSH 2009). A**: Male. **B**: Female. Interpretation of the results for subjects aged ≥75 years requires care due to the small sample size (11 males and 6 females) in the MinaCare data.

As shown in Figure 6A, the proportion of subjects with SBP ≥140 mmHg increased with increasing age both in male and female subjects. A similar trend was seen for the mean SBP values (data not shown). The proportion of subjects with DBP ≥90 mmHg is shown in Figure 6B. In contrast with SBP, the proportion of male subjects with DBP ≥90 mmHg peaked at mid-50s with a value of nearly 20% and declined thereafter. This peak was less pronounced in females, and the proportion seemed to plateau at 60 years or older; the proportion was smaller in females than in males across all age groups. The proportion of subjects in each BP classification category based on the JSH 2009 guideline is shown for each age category in Figure 7 and Table 3. The majority of subjects were classified as “ideal” or “normal” for both sexes in all age groups, and the proportion classified as “ideal” was larger in females than in males. The proportion shifted toward hypertensive classifications with increasing age in both sexes.

**Figure 8:**
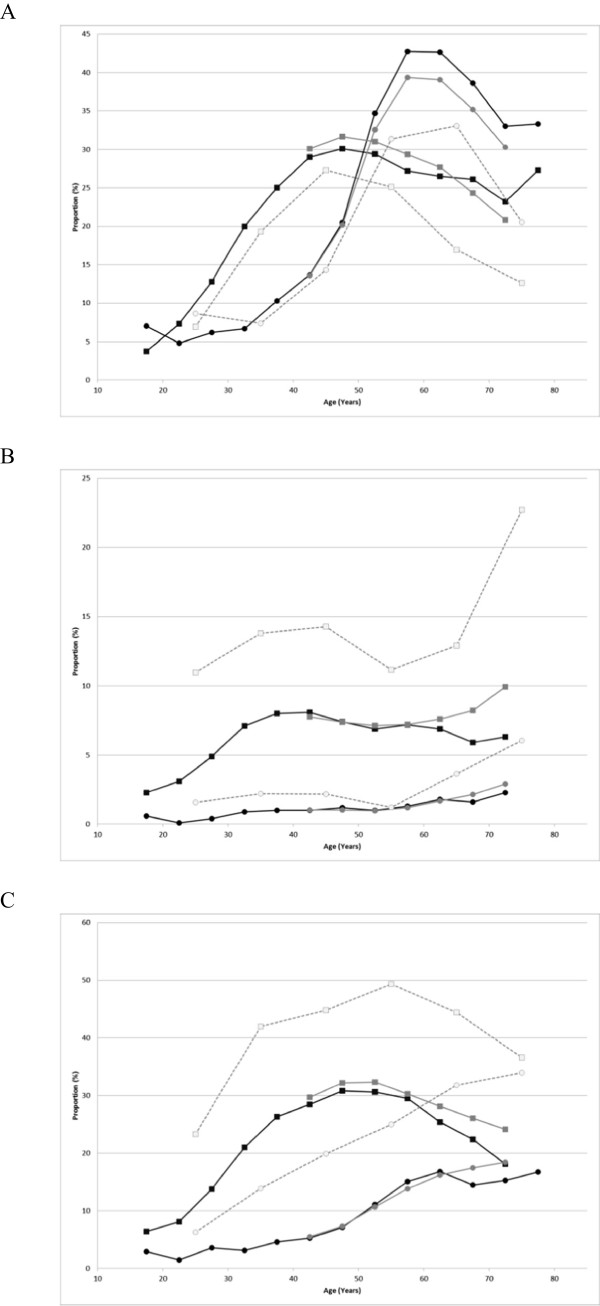
**Lipid parameter distributions in the MinaCare, MHLW-SH, and MHLW-H&N data. A**: Proportions of subjects with LDL-c ≥140 mg/dL. **B**: Proportions of subjects with HDL-c <40 mg/dL. **C**: Proportions of subjects with TG ≥150 mg/dL. Squares represent males and circles represent females. Black, grey, and open symbols represent the MinaCare, MHLW-SH, and MHLW-H&N data, respectively. Interpretation of the result is for subjects aged ≥75 years requires care due to the small sample size (11 males and 6 females) in the MinaCare data.

The proportion of subjects with LDL-c ≥140 mg/dL, HDL-c <40 mg/dL, and TG ≥150 mg/dL are shown in Figures 8A, B, and C, respectively. For LDL-c, male and female subjects showed different patterns for the proportion. In males, the proportion peaked at around 50 years with a value of 30%; but in females, it peaked at a higher age group with higher peak values (above 40%). The proportion was lower in females than in males aged <50 years, but was reversed (higher in females) in higher age groups. The trend was similar for the mean LDL-C values (data not shown). The proportion of females with HDL-c <40 mg/dL was lower than that of males in all age groups; the difference between both sexes increased and peaked around 40 years and narrowed very slightly in higher age groups. A similar trend in the difference between sexes was seen for the mean HDL-c values, which were generally 5–10 mg/dL higher in female subjects (data not shown). The proportion of subjects with TG ≥ 150 mg/dL reached a peak of approximately 30% at 50 years in males, whereas the proportion increased to 60 years and then plateaued in females. The proportions in males were higher across all age groups. A similar trend was seen for the mean TG values (data not shown). Finally, the proportion of subjects with HbA1c ≥ 6.1% showed a generally monotonic increase with increasing age in both male and female subjects, with generally higher values in males. The proportions reached a plateau at a lower age group in males (around 60 years) compared with females. The mean HbA1c values showed similar trends (data not shown). It was noted that the database contained a substantial proportion of missing values for HbA1c (approximately 30% in males and 15% in females). This may have an impact on the interpretation of the results if the missing values did not occur at random (e.g. lower values are more likely to be missing). Similar age trends as those for HbA1c were seen for FBG values.

**Table 3:** **Proportions (%) of subjects in each BP classification category (JSH 2009)**

Sex	Age	BP classification category					
		Ideal	Normal	Normal (high)	HT class I	HT class II	HT class III
Male	<20	55.4	32.5	9.1	2.9	n/a	n/a
	20- < 25	53.8	33.7	9.2	3.0	0.3	n/a
	25- < 30	49.8	34.6	10.3	4.8	0.5	0.0
	30- < 35	47.6	33.1	10.9	6.8	1.4	0.2
	35- < 40	51.7	27.3	12.0	6.8	1.7	0.5
	40- < 45	45.7	26.8	13.8	10.2	2.7	0.8
	45- < 50	40.3	25.7	15.3	13.8	3.8	1.0
	50- < 55	32.4	25.8	18.2	17.8	4.7	1.0
	55- < 60	29.0	25.4	19.6	20.1	5.0	0.9
	60- < 65	28.4	24.4	21.3	19.6	5.6	0.8
	65- < 70	27.7	23.5	23.0	20.9	3.9	0.9
	70- < 75	28.0	22.3	23.8	21.0	4.4	0.5
	75-	45.5	27.3	n/a	27.3	n/a	n/a
Female	<20	83.2	14.5	1.2	1.2	n/a	n/a
	20- < 25	85.6	12.2	1.2	0.9	0.1	n/a
	25- < 30	84.1	13.4	2.1	0.3	0.1	n/a
	30- < 35	81.9	13.8	2.7	1.3	0.3	0.1
	35- < 40	79.8	13.0	4.7	2.0	0.4	0.1
	40- < 45	74.7	14.6	6.3	3.3	0.8	0.3
	45- < 50	64.5	18.1	9.7	5.8	1.6	0.4
	50- < 55	53.9	21.0	13.0	9.4	2.1	0.6
	55- < 60	44.8	23.5	17.0	11.4	2.7	0.6
	60- < 65	38.5	23.4	20.2	14.2	3.0	0.7
	65- < 70	35.1	24.2	21.1	15.9	3.2	0.5
	70- < 75	29.3	24.1	23.4	17.7	4.5	1.0
	75-	33.3	n/a	66.7	n/a	n/a	n/a

JSH2009: The Japanese Society of Hypertension Guidelines for the Management of Hypertension, 2009; HT class: hypertension class; n/a: no subject in the indicated category.

### Comparison with the MHLW-SH and MHLW-H&N data sources

#### Demographics and age distributions

A notable difference was found in the age distribution of male and female subjects among the data sources. The proportion of subjects aged 40–49 years was higher in females than in males in MinaCare data, whereas the proportion was similar between the sexes (MHLW-H&N) or slightly higher in males (MHLW-SH) in the other two data sources. The MinaCare database included data of subjects <40 years, while the MHLW-SH data source did not (Figure 2). Age distribution in the MHLW-H&N data source was more evenly spread in subjects aged from <19 to >70 years compared with the other two data sources, due to its sampling design.

In MHLW-SH data, age distributions for each of the six types of health insurance schemes were available (Figure 3). When we limited subjects to those aged 40 years or older in MinaCare data, age distributions in MinaCare data were most similar to those in employment-based health insurance in MHLW-SH data (see G4 “Union” in Figure 3).

#### BMI and waist circumference

Trends in the proportions of subjects with BMI ≥25 kg/m^2^ with increasing age were similar among the three data sources. In males, the proportion rapidly increased with increasing age up to 40 years, and the highest proportion was found among subjects aged 40–49 years. In females, the proportion gradually increased with increasing age. However, the proportion in MHLW-H&N data was generally higher across all age groups compared with that in MinaCare and MHLW-SH data (Figure 4A). The mean BMI values showed trends similar to those for proportions (data not shown).

The proportion of subjects with a waist circumference ≥85 cm in males and ≥90 cm in females was slightly higher across all age groups in MHLW-H&N data compared with MinaCare and MHLW-SH data (Figure 4B).

#### BP

The BP value was the average of the parameters from two BP measurements, and the same protocol was adopted across the three data sources.

In all data sources, the proportion of subjects with SBP ≥140 mmHg increased with increasing age (Figure 6A); however, the proportions in the MHLW-H&N data source diverged from those in the other two data sources in higher age groups. Across the age range, the proportions were the smallest in MinaCare data, closely followed by the proportions in MHLW-SH data. The highest proportions were found in MHLW-H&N data, and >50% reported SBP ≥140 mmHg in the highest age group for both sexes. In MinaCare data, the proportions remained at values below 30% in all age groups for both sexes. Similar trends were found for the mean SBP values (data not shown).

Similar to SBP, the proportions of DBP ≥90 mmHg were the smallest both in males and females in MinaCare data, followed by the proportions in MHLW-SH data (Figure 6B). Notably higher proportions were seen across all age groups for both sexes in the MHLW-H&N data source compared with the other two data sources.

#### Lipid parameters

The proportion of subjects with LDL-c ≥140 mg/dL in MinaCare and MHLW-SH data was similar across the age groups (Figure 8A). Although the pattern of change with increasing age was similar to that in the other two data sources, the proportions were lower across all age groups in the MHLW-H&N data source when compared with the other two data sources. More than 30% of females had LDL-c ≥140 mg/dL in the age group of 60–69 years in all data sources. A similar trend was found for the mean LDL-c values (data not shown).

The proportion of subjects with HDL-c <40 mg/dL was higher in the MHLW-H&N data source than in the other two data sources, particularly in male subjects (Figure 8B). The proportions increased with increasing age in subjects 60 years or older in all the three data sources, except for male subjects in MinaCare data who showed a slight decline.

The proportion of subjects with TG ≥150 mg/dL showed a similar trend for MinaCare and MHLW-SH data (Figure 8C). Although the pattern of change with increasing age was similar to that in the other two data sources, the proportions were much higher across all age groups in MHLW-H&N data source compared with the other two data sources.

#### HbA1c levels

**Figure 9:**
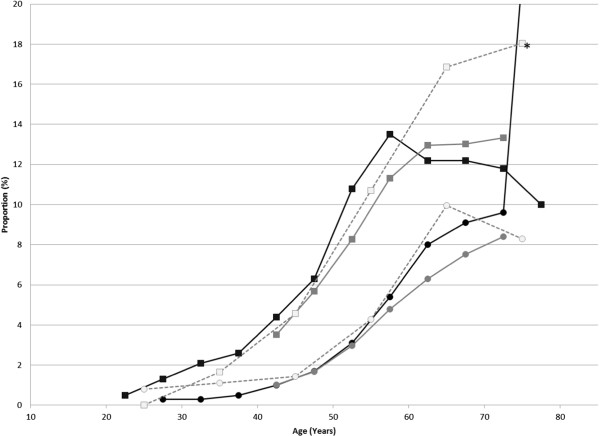
**Distributions of subjects with HbA1c ≥6.1% in the MinaCare, MHLW-SH, and MHLW-H&N data.** Squares represent males and circles represent females. Black, grey, and open symbols represent the MinaCare, MHLW-SH, and MHLW-H&N data, respectively. Interpretation of the result for subjects aged ≥75 years requires care due to the small sample size (11 males and 6 females) in the MinaCare data. * The proportion for females of ≥75 years in the MinaCare data is off-scale.

The proportion of male subjects with HbA1c ≥6.1% showed a generally similar pattern among all the three data sources (Figure 9). In MHLW-H&N data source, the proportions were 5-6% higher than those in the other two data sources in males aged 65–74 years. An apparent small peak was seen at 50–54 years in MinaCare data, but a similar peak was not seen for the mean HbA1c values (data not shown). The proportion of male subjects with HbA1c ≥6.1% was high (33.3%) in females 75 years or older; however, the sample size of subjects was small (6 subjects). The mean HbA1c values were generally similar among the three data sources (data not shown).

## Discussion

In this study, BP, lipid parameters, and blood glucose levels in Japanese residents were investigated using the MinaCare database. Data in the MinaCare database were also compared with those in the two national data sources: MHLW-SH and MHLW-H&N. The results of age trends by sex for the parameters analyzed in this study were generally consistent across the three data sources evaluated in this study. The results of most of the parameters analyzed in this study were also consistent with those reported in previous studies in Japan [13–15].

This Japanese study showed that target BP, LDL-c and HbA1c levels were not achieved in a substantial number of subjects. The proportion of subjects with systolic BP (SBP) of ≥140 mmHg, LDL-C levels of ≥140 mg/dL, and HbA1c levels of ≥6.1% generally increased with increasing age. For example, if one focused on the upper-end age group representing the majority of the MinaCare study population (i.e. age range, 55–59 years), the proportions of subjects with SBP of ≥140 mmHg, LDL–C levels of ≥140 mg/dL, and HbA1c levels of ≥6.1% were 19.0%/12.2% (males/females), 27.2%/ 42.7%, and 13.5%/5.4%, respectively.

As per the World Health Organization (WHO) reports, the prevalence of CVDs has increased worldwide over time, and increased BP, cholesterol and blood sugar levels have been found to be significant health care issue on a global base [16]. According to the WHO’s World map showing the global distribution of the burden of CVDs, Japan is categorized as a country of with a less CVD burden, and it is considered that the Japanese populations has relatively good values for BP, LDL-c, and HbA1c levels [16]. As a result, the average life-span of the Japanese population is the longest in the world. In fact, Japan has a well-established health care system and provides free and equal access to health care service to all citizens. This study provides a critical message that even in a country such as Japan with a highly acclaimed health care system, target BP, LDL-C, and HbA1c levels are not achieved in a substantial number of subjects and that CV risks in the population need to be improved. Considering the currently available wide range of pharmacological intervention for HT, hyperlipidemia, and diabetes mellitus, it is highly important to understand the real world situation for enforcing such interventions to achieve better control and reduce the future prevalence of CVDs. In view of these findings, real world data should be proactively introduced worldwide.

A detailed comparative review of these three data sources was conducted in this study. With respect to demographics and age distributions, the limitation of the MHLW-SH data is that it only included subjects aged 40–74 years; therefore, this data source is not suitable to investigate the health conditions of individuals aged <40 years. Age distribution of MHLW-H&N data was evenly spread out due to the sampling design. However, the number of subjects in this data source was much smaller than that in the MinaCare database for every age group, including the group of subjects aged 70 years or older (Figure 2). Thus, the MinaCare database has a strong advantage in terms of the sample size and age distribution.

Regarding investigational items such as BMI, waist circumference, BP, LDL-c, HDL-C, and TG, the results of the MHLW-H&N data source were different results compared with those of the MHLW-SH and MinaCare data sources, and the latter two data sources generally showed similar results. the results of the MHLW-SH data source, which included individuals from all over Japan with a broad range of occupations, were similar to those of the MinaCare data source. Thus, the difference in the results between MHLW-H&N and the MHLW-SH/MinaCare data sources is not explained by the characteristics of the population in either MinaCare or MHLW-SH data.

The unique tendency of the results shown by in the MHLW-H&N data source seems to be related to the distinctive characteristics of its population. Compared with the other two data sources, the MHLW-H&N data source included individuals who lived in selected areas of every prefecture that had the smallest population. As recently reported, the MHLW-H&N data showed significant bias in the assessment of health status due to limited data, the methods used to identify target subjects, and quality control issues [10, 17]. Dissimilarities between data for BMI, waist circumference, BP, LDL-c, HDL-c and TG in the MHLW-H&N data source and data in the other two data sources could be caused by these biases in the population included in these data sources. Unfortunately, however, we could not further investigate the background of the differences between data in the MHLW-H&N data source and the other two data sources. The reason for this is that access to subject-level data from these national data sources is restricted, which is another limitation of these two data sources. The MinaCare database has high flexibility in analysis of subject-level data, which is a strong advantage for assessing the true meaning of individual values.

The MinaCare database consists of data from corporate employees and their dependents in private corporate health insurance societies. This means that their demographics and socioeconomic status may not represent the overall Japanese population. Elderly subjects are particularly underrepresented in the MinaCare database. Although there are such limitations of the MinaCare database, we believe that the database is a proper reflection of the health status of the people insured by employment-based health insurance, and that it is highly valuable when studying that population.

In summary, the MinaCare database has several strengths including low selection bias due to the high screening rate for annual health checkups among the target population, larger sample size compared with that of MHLW-H&N, and wider age distribution compared with that of MHLW-SH. One remarkable advantage of the MinaCare database is that a longitudinal investigation can be conducted based on subject-level data, which allows investigators to follow up health checkup data from the same individual over time. Another notable advantage is that by combining health checkup data with pharmaceutical claim data that are also available in the database, disease prevalence, intervention outcomes, and the efficacy and safety of treatments can also be studied. Such future studies using the MinaCare database will provide important information to understand and improve the health conditions of the insured population in Japan. Concurrently, disease management processes and methods that measure effects will be globally improved by research on these databases.

## Conclusions

In this study, the distribution of real-world values of BP, LDL-c, and HbA1c were fully described using the MinaCare database. Analysis of MinaCare data indicated that a substantial proportion of subjects did not achieve target BP, LDL-c, and HbA1c levels to reduce the risk of future CVD events even in Japan where health care service is relatively well provided in the world. The results were compared with those from the publicly available reports of MHLW-SH and MHLW-H&N. Age trends by sex for various parameters were generally consistent in the three data sources evaluated in this study, although some notable differences were observed.

Considering the characteristics of the MinaCare database, such as low selection bias, large sample size, wide age distribution, and high flexibility in analysis of subject-level data, the database is highly valuable for studying the health status of the individuals insured by employment-based health insurance.

## Abbreviations

BP: Blood pressure
BMI: Body mass index
CVD: Cardiovascular and cerebrovascular disease
DBP: Diastolic blood pressure
FBG: Fasting blood glucose
HbA1c: Hemoglobin A1c
HDL-c: High-density lipoprotein cholesterol
HT: Hypertension
JDS: Japan diabetes society
JSH: Japanese society of hypertension
LDL-c: Low-density lipoprotein cholesterol
MHLW: Ministry of Health, Labour and Welfare
MHLW-H&N: MHLW national health and nutrition survey
MHLW-SH: MHLW specific health checkups and specific health guidance
n: Number of subjects
NGSP: National glycohemoglobin standardization program
SBP: Systolic blood pressure
SD: Standard deviation
TG: Triglyceride.
